# Supplementary material for: Integrative proteo-genomic profiling uncovers key biomarkers of lapatinib resistance in HER2-positive breast cancer
Source: Br J Cancer. 2025 Sep 13;133(10):1471–82. doi: 10.1038/s41416-025-03174-3 (PMC12603111; doi:10.1038/s41416-025-03174-3)
Supplement: Supplementary file 1 — Supplementary Figure Legends (Figs. 1 and 2) [file 41416_2025_3174_MOESM1_ESM.docx]

**Supplementary Fig 1.** **(A)** Additional fluorescence microscopy images confirming the expression of HER2 protein in both cell types, with secondary antibody only staining (2° only control) used as a control to check if cells are autofluorescence. HER2 protein. 20x magnification. Scale bars: 50μm. Images were analysed using imageJ. Background subtraction was applied using a rolling ball radius of 50 pixels, followed by automatic brightness adjustment. A threshold was set to highlight HER2-positive regions (red), and the ‘analyse particles’ function was used to identify and quantify these regions. Mean fluorescence intensities were extracted, grouped by cell type, and analysed in GraphPad Prism. An unpaired t-test with Welch’s correction (accounting for unequal variances) revealed a significant difference in HER2 expression between groups (p < 0.0001, two-tailed). Data represent individual HER2-positive regions: n = 79 for parental cells and n = 31 for lapatinib-resistant cells. **(B)** The representative insert size distribution demonstrates clear modulation of the signal, with distinct peaks corresponding to both mono-nucleosomes and di-nucleosomes, indicating well-defined nucleosome positioning. **(C)** Representative plot of aggregate signal around transcription start sites (TSS) shows strong enrichment of reads near TSS regions. **(D)** Correlation of chromatin accessibility between different cell lines and across technical replicates. **(E)** MA Plot of differential accessibility (log2 fold change in reads per accessible region) versus the mean number of reads per region, adjusted p value <0.05.

**Supplementary Fig 2. (A)** A comparison of SKBR3 SKBR3-L cell lines based on their significant differential logfold changes with adjusted p value of < 0.05. **(B)** Distance to the nearest transcription start sites (TSSs) for all differentially accessible regions in SKBR3 and SKBR3-L cells (p value of <0.05).
