# Supplementary figures and images for: Integrative proteo-genomic profiling uncovers key biomarkers of lapatinib resistance in HER2-positive breast cancer
Source: Br J Cancer. 2025 Sep 13;133(10):1471–82. doi: 10.1038/s41416-025-03174-3 (PMC12603111; doi:10.1038/s41416-025-03174-3)

A

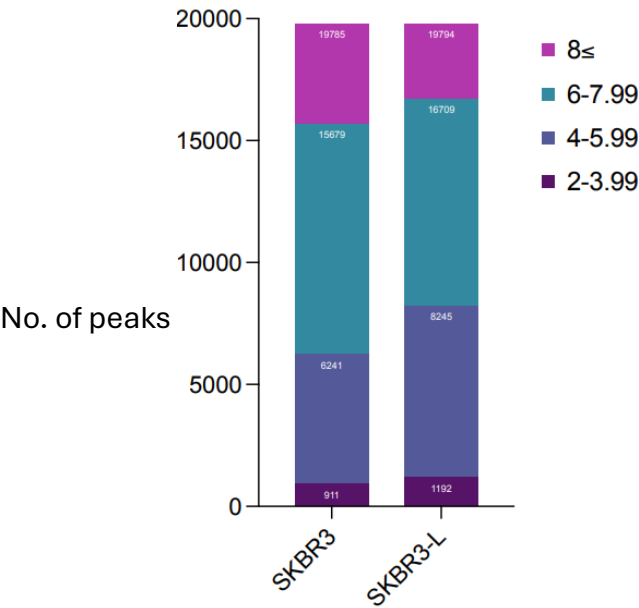

B

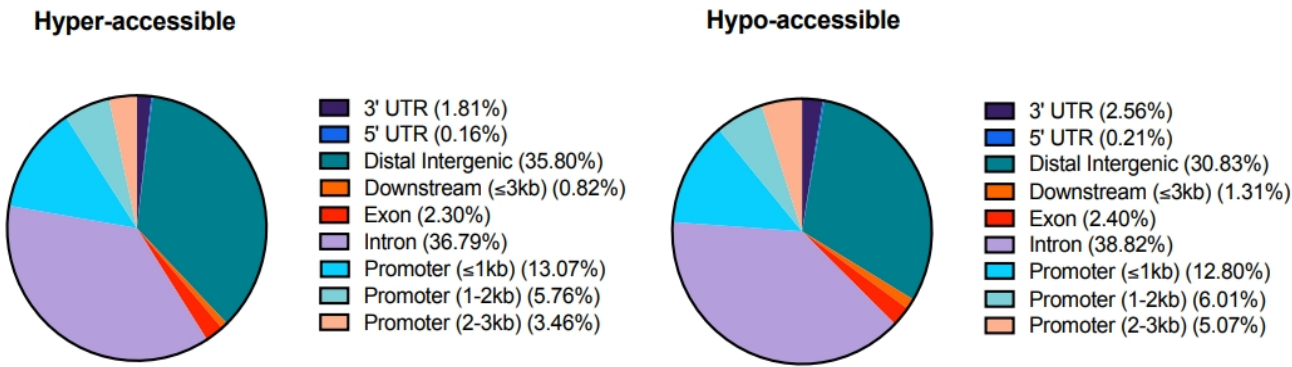

Supplement: Supplementary file 2 — Supplementary Figure 2 [file 41416_2025_3174_MOESM2_ESM.pdf]

A

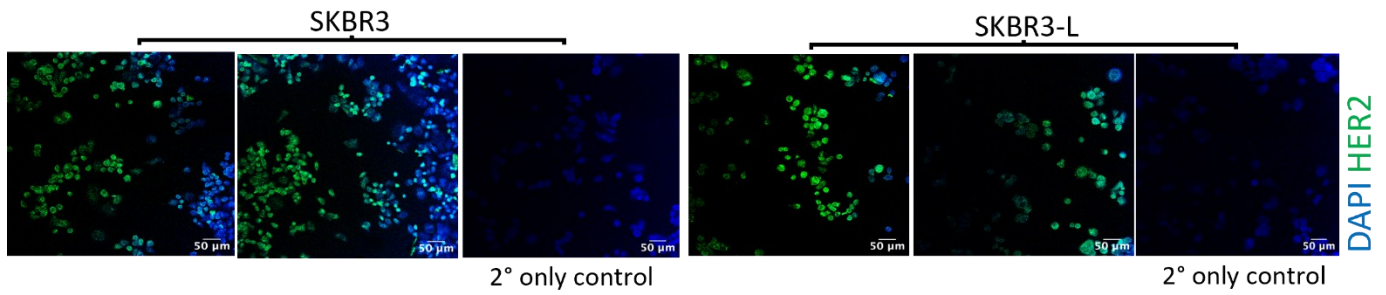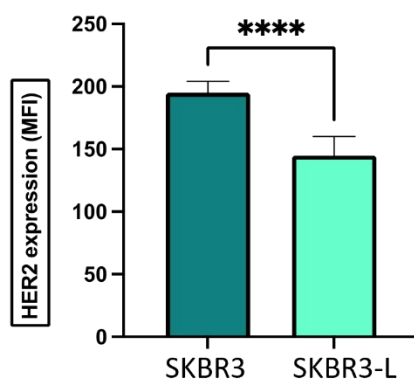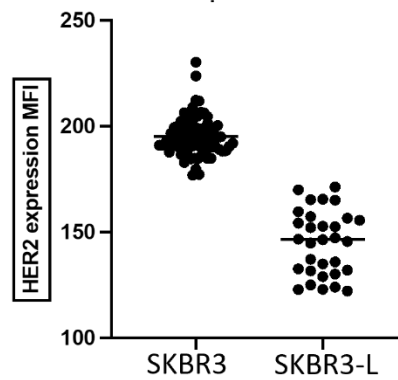

B

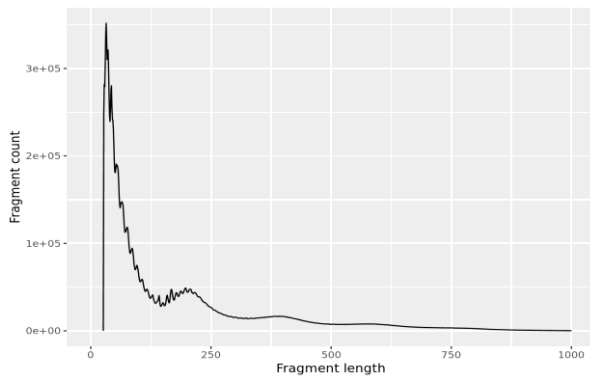

C

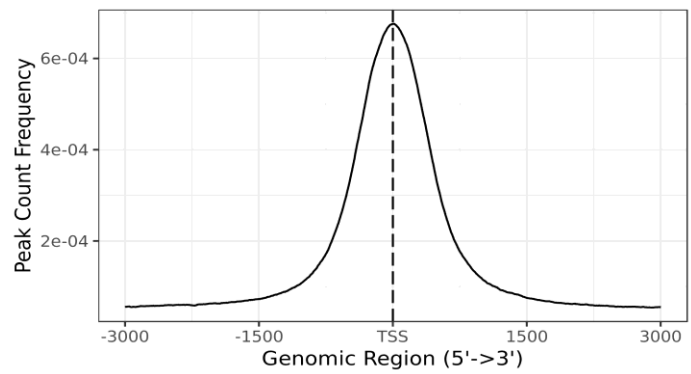

D

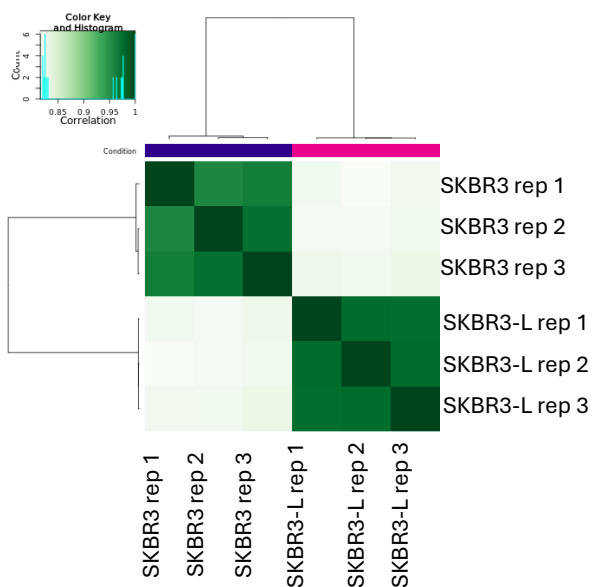

E

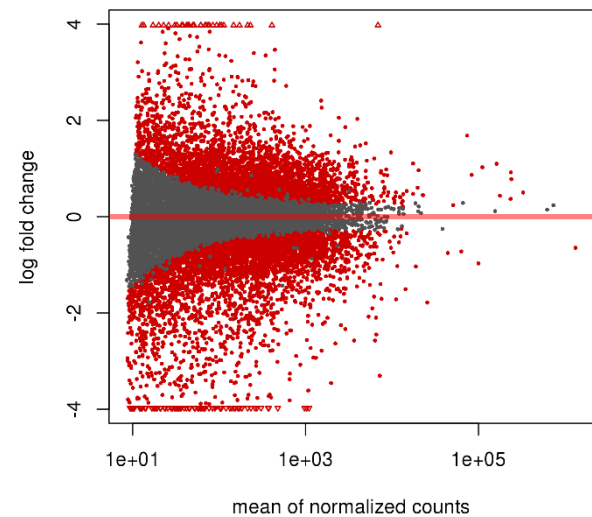

Supplement: Supplementary file 3 — Supplementary Figure 1 [file 41416_2025_3174_MOESM3_ESM.pdf]
